# Understanding Hypertriglyceridemia: Integrating Genetic Insights

**DOI:** 10.3390/genes15020190

**Published:** 2024-01-30

**Authors:** Mara Alves, Francisco Laranjeira, Georgina Correia-da-Silva

**Affiliations:** 1 Faculty of Pharmacy, University of Porto, 4050-313 Porto, Portugal; up202001064@edu.ff.up.pt; 2 CGM—Centro de Genética Médica Jacinto de Magalhães, Centro Hospitalar Universitário de Santo António (CHUdSA), 4099-028 Porto, Portugal; francisco.laranjeira@chporto.min-saude.pt; 3 UMIB—Unit for Multidisciplinary Research in Biomedicine, ICBAS—School of Medicine and Biomedical Sciences, University of Porto, 4050-346 Porto, Portugal; 4 ITR—Laboratory for Integrative and Translational Research in Population Health, 4050-600 Porto, Portugal; 5 UCIBIO Applied Molecular Biosciences Unit and Associate Laboratory i4HB—Institute for Health and Bioeconomy Laboratory of Biochemistry, Department of Biological Sciences, Faculty of Pharmacy, University of Porto, 4050-313 Porto, Portugal

**Keywords:** Hypertriglyceridemia, familial chylomicronemia syndrome, multifactorial chylomicronemia syndrome

## Abstract

Hypertriglyceridemia is an exceptionally complex metabolic disorder characterized by elevated plasma triglycerides associated with an increased risk of acute pancreatitis and cardiovascular diseases such as coronary artery disease. Its phenotype expression is widely heterogeneous and heavily influenced by conditions as obesity, alcohol consumption, or metabolic syndromes. Looking into the genetic underpinnings of hypertriglyceridemia, this review focuses on the genetic variants in *LPL*, *APOA5*, *APOC2*, *GPIHBP1* and *LMF1* triglyceride-regulating genes reportedly associated with abnormal genetic transcription and the translation of proteins participating in triglyceride-rich lipoprotein metabolism. Hypertriglyceridemia resulting from such genetic abnormalities can be categorized as monogenic or polygenic. Monogenic hypertriglyceridemia, also known as familial chylomicronemia syndrome, is caused by homozygous or compound heterozygous pathogenic variants in the five canonical genes. Polygenic hypertriglyceridemia, also known as multifactorial chylomicronemia syndrome in extreme cases of hypertriglyceridemia, is caused by heterozygous pathogenic genetic variants with variable penetrance affecting the canonical genes, and a set of common non-pathogenic genetic variants (polymorphisms, using the former nomenclature) with well-established association with elevated triglyceride levels. We further address recent progress in triglyceride-lowering treatments. Understanding the genetic basis of hypertriglyceridemia opens new translational opportunities in the scope of genetic screening and the development of novel therapies.

## 1. Introduction

Hypertriglyceridemia (HTG) is a metabolic disorder characterized by elevated plasma triglyceride (TG) levels above an established threshold value that varies among international expert groups [1]. Normotriglyceridemia, as acknowledged by consensus committees like the European Society of Cardiology and the European Atherosclerosis Society, is characterized by a fasting plasma TG concentration below 1.7 mmol/L or 150 mg/dL [2]. HTG displays an approximate prevalence of 10% in the global adult population with considerable inter-regional variability possibly related to environmental factors and genetic background [3,4]. HTG is associated with an increased risk of acute pancreatitis (AP) and cardiovascular disease (CVD) such as coronary artery disease [2,5]. Plasma TG concentration is determined by the intricate interplay of genetic and non-genetic factors. The genetic framework of numerous common and rare variants (with frequencies above and below 1%, respectively) within TG-associated *loci* may partially account for the broad spectrum of lipid phenotypes, ranging from normotriglyceridemia to the most severe cases of HTG [6,7].

HTG can be classified according to its biochemical phenotype (TG concentration) as mild-to-moderate HTG and severe HTG, and according to its genotype, as monogenic HTG or polygenic HTG [8,9]. Regarding phenotypic expression, it can be defined as mild-to-moderate and severe HTG given ranges of 2.0–9.9 mmol/L (175–885 mg/dL) and ≥10 mmol/L (≥885 mg/dL), respectively [10].

Total plasma TG concentration comprises the TG content of multiple lipoprotein particles. The traditional lipid panel measures the plasma TG within all lipoproteins, predominantly in triglyceride-rich lipoproteins (TRLs), chylomicrons (CMs), and very-low-density lipoproteins (VLDLs). CMs are produced in enterocytes from dietary lipids carrying the apolipoprotein B-48 (apoB-48). After entry into the systemic circulation, CMs are progressively hydrolyzed in capillary beds by the action of the enzyme lipoprotein lipase (LPL) to Chylomicron remnant (CR) particles that undergo hepatic clearance. VLDL particles are hepatically synthesized TRLs composed of apoB-100, TGs, and cholesteryl esters and secreted into the systemic circulation.

As generally accepted, mild-to-moderate HTG primarily reflects the accumulation of VLDLs and related remnant particles in the plasma, while severe HTG usually indicates the presence of CMs under fasting conditions plus excess VLDLs and related remnants [9,10]. HTG may arise as result of the defective metabolism of TRLs and their remnants, i.e., impaired clearance, increased production, or both. Considering the unique genetic archetype of each individual, the manifestation of HTG can originate from either a monogenic or polygenic basis. Familial chylomicronemia syndrome (FCS) is a rare form of monogenic HTG with rare, biallelic (homozygous or compound heterozygous) variants in genes encoding proteins involved in the metabolism of TRLs. Monogenic phenotype expression is associated with the development of severe HTG, regardless of existing secondary factors [9,11,12]. On the other hand, polygenic HTG is characterized by heterozygous high-effect variants in canonical genes involved in TRL metabolism and a high burden of common low-effect polymorphisms in candidate genes associated with elevated plasma TG. The phenotype is widely heterogeneous, ranging from normolipidemia to severe HTG, being influenced by environmental and lifestyle factors [9,10,13]. The secondary factors predisposing one to HTG include biological traits (age, sex, and ethnicity), lifestyle habits (smoking, excessive alcohol consumption, sedentary lifestyle, and stress), underlying diseases (diabetes, obesity, and hypothyroidism), and medication, among other factors [2,5,8]. Therefore, the phenotypic expression of HTG results from the cumulative burden of genetic and non-genetic factors [2,14,15].

## 2. Familial Chylomicronemia Syndrome

FCS, monogenic chylomicronemia, monogenic hypertriglyceridemia, traditionally also known as hyperlipoproteinemia type 1 (MIM#238600), is a metabolic disorder of autosomal recessive inheritance characterized by severe HTG [7,8,9,10,16]. The overall population prevalence of FCS is reported to be quite rare, with published estimates of approximately one to 10 in every 1,000,000 people [8,17]. FCS patients manifest the disease mainly during childhood and adolescence [10]. The monogenic phenotype is caused by biallelic pathogenic variants (homozygous or compound heterozygous) in genes regulating TRL catabolism [16,18,19]. The severity of HTG is largely dependent on the activity of LPL. More than 90% of cases of monogenic chylomicronemia are caused by biallelic loss-of-function (LOF) pathogenic variants in the LPL encoding gene, the *LPL* gene [2,8]. The remaining biallelic LOF variants associated with FCS predominantly occur in genes encoding proteins involved in the lipolytic process, assembly and transport of the LPL enzyme, namely apolipoprotein C-II (*APOC2* gene), apolipoprotein A-V (*APOA5* gene), lipase maturation factor 1 (*LMF1* gene), and glycosylphosphatidylinositol-anchored high-density lipoprotein-binding protein 1 (GPIHBP1) (*GPIHBP1* gene) [7,18,20]. Biallelic pathogenic variants in these five canonical genes (*LPL*, *APOC2*, *APOA5*, *LMF1* and *GPIHBP1*) drastically disrupt LPL lipolytic activity and therefore CM lipolysis and clearance, causing chylomicronemia, which is a CM persistence in the plasma beyond the fasting window of 12 to 14 h [8,9,11]. A cohort study of 52 patients affected by the condition compared phenotypes between FCS caused by pathogenic variants in *LPL* and in non-*LPL* genes. Around 80% carried biallelic pathogenic variants in the *LPL* [12]. Of the remaining individuals, almost half carried biallelic pathogenic variants in the *GBPIHBP1* gene, and the others carried homozygous/compound heterozygous pathogenic variants in the *APOC2*, *APOA5* and *LMF1* genes, or double heterozygous pathogenic variants with one normal *LPL* allele. These molecular subtypes were largely phenotypically similar with severe HTG. A more recent study conducted molecular genetic testing of DNA samples from 459 unrelated hypertriglyceridemic subjects to identify the genetic cause of severe HTG [21]. Of the 459 patients, 356 (77.6%) were males and the mean (±SD) age at the time of genetic testing was 42.6 (±11.2) years. Four of the 459 patients (0.9%) had monogenic HTG: two patients homozygous for *GPIHBP1* pathogenic variants, one patient homozygous for an *LPL* variant, and one patient compound heterozygous for *LPL* variants. These data suggest that there is a strong polygenic basis for severe HTG in a large proportion of patients.

The pathological persistence of CMs in the circulation induces complications that contribute to increasing morbidity and mortality [11,16,19]. The recurrence of acute pancreatitis episodes is the most distressing clinical manifestation, with an increased risk of progression to chronic pancreatitis and necrosis of the pancreatic tissue [11] and exocrine pancreatic insufficiency as a direct consequence of chronic pancreatitis [22]. Other symptoms include abdominal pain, eruptive xanthomas of the trunk and limbs, lipemia retinalis, hepatosplenomegaly, nausea, vomiting, fatigue, arthralgias, developmental impairment, and neurological symptoms, which affect quality of life [11,16,19].

## 3. Multifactorial Chylomicronemia Syndrome

MCM (MIM#144650), also known as hyperlipoproteinemia type 5, is a complex condition of oligogenic or polygenic nature, whose phenotypic expression is exacerbated by the presence of secondary non-genetic factors [8,17]. Unlike FCS, MCM manifests mainly throughout adulthood and is considerably more frequent, with an estimated prevalence of one in every 600 people [6,23]. Pancreatitis is less frequent than in FCS, but the clinical features associated with MCM encompass those linked to chylomicronemia. There are essentially two subtypes of predisposing genetic factors: rare heterozygous variants in one of the five canonical genes, and a high burden of common polymorphisms associated with high plasma TG concentration, as reported by genome-wide association studies (GWAS) [7,8]. Common variants collectively lead to a condition of predisposition for HTG onset. The susceptibility induced by these common variants can interact synergistically with rare heterozygous variants in canonical genes and/or non-genetic factors originating the expression of an extreme phenotype, such as severe HTG [9,18]. On the other hand, individuals with normotriglyceridemia may harbor genetic determinants that are disproportionately represented in HTG individuals. Therefore, the presence of such genetic determinants does not guarantee the expression of the HTG phenotype [7,18].

Several studies demonstrated that the heterozygous state for LPL deficiency is associated with a broad heterogeneous phenotype that ranges from normotriglyceridemia to severe HTG. Babirak et al. studied six families of probands with LPL deficiency to characterize the heterozygote state [24]. Despite genotyping not being conducted, since the study was carried out before the genomic era, a careful examination of the phenotype indicated that probands were homozygous for *LPL* variants. A total of thirteen obligate heterozygotes from these families exhibited a widely heterogeneous phenotype, with more than half having normal TG levels, a few having mild HTG, and two having TG > 7 mmol/L. The measurement of LPL activity and mass showed significantly variable results, with one subject having even higher LPL activity compared to the normotriglyceridemic control group. Similarly, Julien et al. found that plasma TG levels ranged from normal to severe HTG among carriers of heterozygous *LPL* variants, highlighting abdominal obesity and fasting hyperinsulinemia as being significantly correlated with HTG [25].

The extent to which rare heterozygous variants in HTG-associated *loci* could contribute to the heritability of HTG was explored by genomic sequencing analysis studies. Johansen et al. demonstrated an increase in the prevalence of heterozygous rare variants within identified candidate genes among hypertriglyceridemic individuals [26]. Most participants were found to have only a single rare variant, but there was a notable overrepresentation of individuals with HTG who possessed multiple rare variants. These findings supported a cumulative burden of rare variants in both known and novel genes in polygenic HTG. In other words, individuals with severe HTG were confirmed to have an excessive prevalence of heterozygous variants in canonical genes *LPL*, *APOC2*, *GPIHBP1*, *APOA5* and *LMF1*, once again indicating that the heterozygous state is a predisposing factor for the development of HTG [27]. An in-depth, systematic evaluation of the genetic determinants of patients affected by severe HTG was conducted. The *LPL*, *APOC2*, *GPIHBP1*, *APOA5*, and *LMF1* genes were screened for rare variants, and a polygenic risk score (PRS) was used to assess the accumulation of common variants. The predominant feature was an extreme accumulation of common variants (high PRS), whereas a substantial proportion of patients also carried heterozygous rare variants. Overall, 46.3% of patients had polygenic HTG, whereas only 1.1% had biallelic or homozygous monogenic HTG [3]. More recently, an evaluation of the longitudinal TG phenotype of individuals heterozygous for pathogenic *LPL* variants was carried out [28]. Plasma TG levels exhibited considerable variation. The majority of patients oscillated between mild-to-moderate and severe HTG and the heterozygous LPL-deficient phenotype was highly variable both within and between patients. It was concluded that heterozygosity confers susceptibility to a wide range of TG phenotypes, with severity likely depending on secondary factors.

Based on these studies, the substantial overrepresentation of rare heterozygous variants within canonical genes in hypertriglyceridemic subjects strongly implies their involvement in predisposing individuals to disease. HTG susceptibility and phenotypic heterogeneity are both influenced by the accumulation of common and rare TG-associated variants [29]. However, the intriguing aspect is that some heterozygous carriers of these variants exhibit a normal lipid phenotype. This observation underscores rare heterozygous variants as partially or incompletely penetrant and carriers can manifest a broad spectrum of phenotypes, ranging from normal to HTG. It is important to emphasize that the mere presence of these variants does not guarantee the expression of a hypertriglyceridemic phenotype, highlighting the complex link between TG-associated genetic variants and lipid outcomes [1,9].

GWA studies have provided a revolutionary contribution in the identification of genetic *loci* harboring common variants with frequencies >1% (single nucleotide polymorphisms, SNP) statistically associated with the heritability of complex phenotypic traits, including plasma lipid concentration [6,7]. A study involving >100,000 individuals with multiple lipid and CVD phenotypes reported 95 *loci* significantly associated not only with normal variation in lipid traits but also with extreme lipid phenotypes [30]. The cumulative effect of GWAS-identified common variants across the TG-associated *loci* accounts for 10% of plasma TG concentration variability, which represents 25–30% of the overall genetic variance.

The genetic architecture of GWAS-identified common polymorphisms could underlie predisposition to HTG [6,7,9,10]. A study that assessed the correlation between common variants linked to mild TG variations identified in GWA studies and their association with severe HTG revealed substantial contributions from the same common variants present in several genes, including *APOA5*, *APOE*, *GCKR*, *TRIB1*, and *TBL2*/*MLXIPL*. Moreover, these common variants were found to be associated with a significant fraction—approximately one-quarter—of the explained variation in disease status [31]. Johansen et al. also constructed weighted allelic risk scores to evaluate the combined impact of common variants associated with lipid levels [29]. Weighted TG risk scores were considerably higher in HTG patients compared to control subjects. Subjects with the highest risk score exhibited a 4.15-fold greater likelihood of being HTG cases than healthy controls, compared with the median risk score bin. Individuals in the highest risk score bin were 23.0 times more likely to be HTG cases than healthy controls compared with subjects in the lowest risk score bin as the reference group.

A cohort of individuals with severe HTG revealed that 32.0% of HTG patients exhibited an elevated polygenic score characterized by TG-raising common variants in 16 *loci*. Conversely, only 9.5% of individuals within the normolipidemic control group demonstrated a similar high polygenic score [3]. Similarly to what was observed with rare heterozygous variants, an elevated risk posed by an enrichment of common variants across GWAS-discovered TG-associated *loci* is significantly associated with HTG, implying that a high PRS could incrementally contribute to HTG susceptibility. Nonetheless, individuals exhibiting normal lipid profiles may also possess an elevated PRS, indicating that a high risk does not assure the phenotypic expression of HTG [29].

In summary, based on the results reported by several studies, both common and rare variants within TG-associated *loci* are substantially overrepresented in hypertriglyceridemic individuals and appear to underlie predisposition to the development of HTG [26,29]. Nevertheless, numerous cases were described where phenotypically normal subjects exhibit an HTG-predisposing genotype, characterized by the accumulation of common and/or rare variants [3,31]. These facts strongly imply that an increased background of partially penetrant variants alone is insufficient to induce the phenotypic expression of HTG. Moreover, the substantial overlap of risk alleles between HTG patients and controls indicates that genetic variants only partially contribute to the interpopulation variability observed in the lipid phenotype [6,9].

## 4. Lipoprotein Lipase

LPL breaks down TG carried by TRLs and the released fatty acids are used as energy or stored in fatty tissue for later use. LPL is predominantly expressed by tissues relying on fatty acids for energy, namely cardiac, muscle, and adipose tissue, where the enzyme is bound throughout the vascular network [32,33,34]. There has been intense focus on the molecular details of LPL and the factors that affect its activity, as many cases of HTG appear to be directly or indirectly related to abnormalities in LPL function [35].

The *LPL* gene is located on chromosome 8q22 and contains ten exons. The mature LPL protein comprises 448 amino acids [36]. LPL contains an N-terminal domain and a C-terminal domain connected by a hinge region. The N-catalytic domain presents a characteristic lipase-specific α/β-hydrolase fold that harbors a serine protease-like catalytic triad, a mobile helical surface which presumably can adopt an open or closed conformation to control substrate accessibility to the active site [33,37]. The lid sequence is contained between two conserved cysteines, which form one of the four disulfide bonds and may provide stabilization of the LPL protein during catalytic activity [36]. In addition, the N-terminal domain has several amino acids involved in the modulation of the calcium cofactor [38]. The C-terminal domain contains a flat β-barrel region notably enriched in tryptophan, a zone for the recognition of lipid substrate [33]. The interaction between the enzyme and the lipoprotein triggers a conformational change, and the enzyme catalytic site is exposed and becomes available to initiate TG hydrolysis.

The LPL surface lipophilicity acquired by hydrophilic residues in the N-terminal lid and C-terminal lipid-binding regions forms hydrophobic patches that may facilitate TG access to the catalytic cleft [39]. A basic patch that spans throughout the N- and C-terminal domains of LPL interacts with the negatively charged extracellular HSPG and the acidic domain of GPIHBP1 [40]. Prior to entering the secretory pathway, the nascent LPL protein undergoes post-translational modifications in the ER that allow for the proper folding and catalytic activity of the mature LPL protein. N-glycosylation and three N-terminal disulfide bonds are considered essential for the activity [33]. It was proposed that a head-to-tail homodimer formation was critical for LPL secretion and activity, though structural works have challenged this paradigm, proposing that LPL preserves catalytic activity as a monomer [32,33]. Moreover, it is necessary for LPL to interact with GPIHBP1 to maintain stability, and this complex functionality was demonstrated by several studies [39]. More recently, Gunn and Neher succeeded in using cryogenic electron microscopy to solve the structure of a dimeric LPL oligomer. They also observed a hydrophobic pore adjacent to the LPL active site. As suggested by the authors, this could provide additional ligand specificity beyond the lid peptide and the hydrophobic binding pocket [41]. Beigneux et al. proved that LPL and GPIHBP1-bound LPL are active in a monomeric state and that monomers of LPL may be active in the absence of GPIHBP1 [42]. In addition, Arora et al. reported an X-ray crystal structure of LPL in complex with GPIHBP1. The structures and additional biochemical data were consistent with the idea that LPL, in complex with GPIHBP1, can be active as a monomeric 1:1 complex [43].

Genetic studies report over 100 LOF and functionally heterogeneous variants (HGMD: https://www.hgmd.cf.ac.uk, accessed on 15 November 2023) in *LPL* (Table 1) associated with the development of LPL deficiency (MIM*609708) in subjects with FCS [38]. The missense variants c.106G>A (p.Asp36Asn), c.644G>A (p.Gly215Glu), c.701C>T (p.Pro234Leu), c.829G>A (p.Asp277Asn), c.953A>G (p.Asn318Ser), and c.1421C>A (p.Ser474*) are the most commonly reported in *LPL*, showing an allele frequency variation within population groups [44,45]. In a large population study, near-complete linkage disequilibrium was observed between p.Asp36Asn and c.-188-93T>G, a variant affecting the promoter of the *LPL* gene. Carriers of c.-188-93T>G had significantly lower TG levels, while carriers of both variants, c.-188-93T>G and p.Asp36Asn, had the highest TG levels. Carriers of the c.-188-93G variant allele were “protected” against the TG-raising effect of obesity. In vitro functional assays in a rat smooth muscle cell line and in a human adrenal cell line confirmed increased promotor activity of the c.-188-93G variant compared with c.-188-93T wild-type allele [46]. Several variants have also been associated with altered HDL-C concentrations [47]. The variant with substitution of glutamic acid for glycine in exon 5 occurs within the longest segment of homology for the LPL protein amongst different species and results in a catalytically defective protein [48]. Greater knowledge of the underlying mechanisms of these variations within the LPL gene may be of considerable importance in understanding genetic predisposition to CVD [49]. In almost all case-control studies, the frequency of the p.Asn318Ser variant carriers is higher in hypertriglyceridemic individuals compared to normolipidemic controls [44].

Polymorphism c.1421C>A (p.Ser474*) has an allele frequency between 12% and 16% [38]. Its expression causes the formation of a truncated protein where the last two amino acids are missing which exhibits a gain-of-function (GOF) phenotype, characterized by decreased plasma TG concentration, increased LPL activity and reduced CVD risk [38,45]. Experimental models have demonstrated that adenoviral-mediated expression of the p.Ser474* variant prevents perinatal mortality in LPL knockout mice [50] and mitigates the phenotype associated with LPL deficiency in patients with FCS [51]. MicroRNAs (miRs) exert post-transcriptional down-regulation and their target sequence on the 3′UTR may be altered by SNPs. The regulation of LPL by several miRs that can be lost in the presence of specific LPL TG-lowering haplotypes has been reported. Consequently, p.Ser474* association with TG concentration could be at least partially explained by its strong linkage disequilibrium with these functional 3′UTR SNPs [52].

The loss of the last two amino acids can also enhance the electrostatic interaction between LPL and GPIHBP1, thereby increasing the structural stability of LPL [38]. In a study with five unrelated HTG-AP patients heterozygous for the rare East Asian-specific missense variant c.862G>A (p.Ala288Thr), the LPL functional activity was analyzed. All patients were overweight adult males with a long history of alcohol consumption. The variant caused only a mild effect on LPL functional activity with an approximate 20% reduction of LPL protein secretion compared to wild type. The combination of p.Ala288Thr heterozygosity with alcoholism may have triggered HTG onset [53].

Variants localized within the intronic region, particularly at the intron-exon junction, severely compromise mRNA processing, intron splicing, and the nature and number of protein products synthesized. Variants in the 5′ and 3′-UTR sequences composed of regulatory elements can positively or negatively influence gene expression [37]. The variant c.1322+1G>A is a splice site donor variant in the first nucleotide of intron 8 that leads to the loss of the donor splice site followed by aberrant splicing of the *LPL* mRNA and the formation of alternative transcripts [54]. Another variant c.-188-39T>C induces a change in the promoter element complementary to the transcription factor Oct-1 and results in highly reduced promoter activity [37].

The majority of genetic variants responsible for FCS onset and manifestation occur in the codifying regions and are found in exons 5 and 6, therefore interfering with the secretion, stability and catalytic activity of the enzyme [45]. Exon 5 encodes for the lid structure and part of the region that binds to TG. Exon 6 encodes for two structurally important disulfide bonds as well as two positively charged amino acid complexes which bind to heparin [37,55]. Missense variant c.209A>G (p.Asn70Ser) severely compromises the N-glycosylation of residue 43 [56]. Missense variants c.615T>A (p.Cys243Ser) [37] and c.798C>G (p.Cys266Trp) lead to the loss of disulfide bond-forming cysteines [57]. Variants compromising amino acids that constitute the catalytic triad (c.547G>A [p.Asp183Asn], c.548A>G [p.Asp183Gly], c.547G>C [p.Asp183His]), or in close proximity to the catalytic site (c.542G>A [p.Gly181Ser], c.809G>A [p.Arg270His], c.809G>T [p.Arg270Leu]) significantly reduce catalytic activity [37]. Other variants like c.1306G>A (p.Gly436Arg) and c.1310A>T (p.Glu437Val) increase LPL susceptibility to the endoproteolytic cleavage at residue 297 (a known furin protease cleavage site) [58].

## 5. Apolipoprotein C-II

Apolipoprotein C-II (apoC-II) is a small exchangeable apolipoprotein that plays a critical role in the metabolism of TRLs by acting as a cofactor of LPL, enhancing LPL catalytic activity. This apolipoprotein is primarily expressed in the liver and secreted into plasma, but is also produced by other tissues including the intestine and adipose tissue [45,59,60].

Apolipoprotein C-II is encoded by the *APOC2* gene localized in the *APOE*-*APOC1*-*APOC4*-*APOC2* gene cluster on chromosome 19q13.2 [59]. Upon expression of the *APOC2* gene, the newly synthetized signal peptide is cleaved and the mature apolipoprotein composed of 79 amino acids is formed. The tertiary structure presents three helices. The N-terminal region located in the first helix is rich in hydrophobic amino acids and responsible for the binding of lipoproteins. The C-terminal region located in the third helix is important for LPL activation and residues Tyr63, Ile66, Asp69, and Gln70 (by the recommended HGVS nomenclature: Tyr85, Ile88, Asp91, and Gln92) form a binding site for the LPL enzyme [45,59,61]. In addition, the C-terminal region may be involved in the conformational change of the lid in order to expose the enzymatic catalytic cleft and allow for the accessibility of TG [59].

ApoC-II deficiency (MIM#207750), also known as SQF, closely simulates LPL deficiency. It is an autosomal recessive disease caused by LOF pathogenic variants (Table 2) in *APOC2* [7,62,63]. There are less than 30 *APOC2* genetic variants published in the literature (HGMD: https://www.hgmd.cf.ac.uk, accessed on 20 December 2023), comprising missense, nonsense, splicing, frameshift and changes in regulatory regions [7,59]. *APOC2* variants induce the expression of a structural unstable transcript susceptible to intracellular degradation. Consequently, carriers exhibit a decreased or undetectable concentration of plasma apoC-II, as well as the drastic reduction or absence of LPL catalytic activity [64,65,66,67]. Apolipoprotein C-II deficiency in newborns can cause severe mental development problems secondary to lipid encephalopathy [67]. The majority of homozygous carriers of *APOC2* variants arise in consanguinity kindreds [64,68,69,70,71,72]. *APOC2* variants include apoC-II_Auckland_ (c.255C>A [p.Tyr85*]) [73], apoC-II_Bari_ (c.177C>G [p.Tyr59*]) [74], apoC-II_Colombia_ (c.133_134del [p.Ser45Glnfs*24]) [75], apoC-II_Nijmegen_ (c.118del [p.Val40*]) [76], apoC-II_Padova_ (c.177C>A [p.Tyr59*]) [68], apoC-II_Shangai_ (c.86delinsCC [p.Asn29Alafs*2]) [77], apoC-II_Toronto_ (c.270del [p.Asp69Thrfs*7]) [78], and apoC-II_Paris2_ (c.10C>T [p.Arg4*]) [79].

ApoC-II_Paris1_ (c.1A>G [p.?]) originates from an A to G transition and the subsequent substitution of initiation codon AUG (methionine) for a GUG (valine) [65]. In transcripts containing the Paris_1_ initiation codon variant (GUG) results in abnormal translation initiation. The analysis of the codifying region of apoC-II_Paris1_ gene downstream of the mutation reveals that the first inframe methionine codon is present at residue +9 of mature apolipoprotein C-II. Translation initiation at this site would eliminate the entire signal peptide as well as the first eight amino acids of the N-terminal functional domain; therefore, the secretion of apoprotein C-II into plasma would be highly unlikely.

ApoC-II_Hamburg_ (c.55+1G>C) [80] and apoC-II_Tokyo_ (c.55+1G>C) [81] result from a single nucleotide substitution, G to C transversion, at 5′donor splice site of intron 2, causing pre-mRNA aberrant splicing and skipping of exon 2, which encodes the initiation codon. The carrier of apoC-II_Hamburg_ had ~14% of detectable hepatic *APOC2* mRNA compared to non-carrier control subjects, due to the fact that aberrant spliced mRNA is frequently unstable and susceptible to intracellular degradation.

ApoC-II_Tuzla_ (g.17,719,326–17,722,303del) [70] occurs from a deletion, which covers the entire coding sequence corresponding to exons 2, 3 and 4. Concerning point variants within the promoter region, the A to G transition in the *APOC2* proximal promoter (c.-25-90A>G) resulted in a substantially diminished binding of the complementary cis-regulatory element, and therefore compromises the transcription [72]. Similarly, T to A transversion at position -190 in the promoter region (c.-25-190T>A) also reduces the transcriptional activity [66].

## 6. Apolipoprotein C-III

Apolipoprotein C-III (apoC-III) is a key regulator of the metabolism of TRLs and plasma TG homeostasis via the inhibition of LPL activity, suppression of hepatic LRT clearance, and modulation of hepatic VLDL particle assimilation and secretion [82,83,84]. Mutations (Table 3) are associated with low plasma TG levels and the reduced risk of CVD. Plasma apoC-III concentration in healthy individuals corresponds to 7–12 mg/dL, while concentrations over 30 mg/dL are frequent in hypertriglyceridemic patients.

The *APOC3 locus* encoding for apoC-III is located in the *APOA4*-*APOC3*-*APOA1* cluster on chromosome 11q23. *APOC3* comprises three intronic regions and four exonic regions [82]. Expression of the *APOC3* gene is promoted by glucose via activation of the transcription factors Carbohydrate response element-binding protein (ChREBP) and HNF-4α. On the other hand, factors such as insulin, PPARα, Rev-erb and FXR inhibit *APOC3* promoter activity [83].

Genetic expression of *APOC3* mainly occurs in hepatocytes and enterocytes. After signal peptide cleavage, a mature protein composed of 79 amino acid residues is formed [82]. The N-terminal domain and the central region are composed of negatively and positively charged residues describing a G-type amphipathic helix. The C-terminal domain has an apolar phase with eight hydrophobic residues, positively charged residues at the polar/apolar interface, and negatively charged residues in the polar phase [85]. The C-terminal region is essential to LPL inactivation. ApoC-III variants lacking hydrophobic residues have lower LPL inhibitory capacity [86].

Studies have demonstrated that higher apoC-III plasma concentration is significantly correlated with elevated plasma TG and an increased risk of atherogenesis development [87]. In contrast, carriers of LOF variants in the *APOC3* gene (MIM*107720) had low serum TG values, as well as a cardioprotective lipid profile [88]. The role of apoC-III in the development of HTG was also verified in experimental models with transgenic rabbits [89]. Overexpression of human apoC-III in the liver of transgenic rabbits triggered severe HTG onset, with higher plasma TG in postprandial and fasting periods compared to non-transgenic animals. The increase in plasma TG levels was attributed to overproduction and reduced clearance of particles within the CM and VLDL density range.

The c.*40G>C polymorphism denominated as *Sst*l (rs5128), which was the first to be identified in the *APOC3 locus*, corresponding to a guanine-to-cytosine transversion in the 3′UTR non-coding region, represents the rare S2 allele, while the wild-type allele represents the most frequent S1 allele. Studies have demonstrated that heterozygous carriers of the S2 allele have high plasma concentrations of apoC-III, as well as an increased risk of developing HTG and CVD [83,90]. Evaluation of the hepatic expression of *APOC3* mRNA in heterozygous carriers of the S2 allele and homozygous S1 controls demonstrated overexpression compared to the control group [91]. The process by which the S2 allele induces overexpression of the *APOC3* gene remains poorly understood. Given its location in the functional 3′UTR region, the polymorphism may be involved in regulating gene expression or mRNA stabilization. The S2 allele can also act as a marker for other functional variants within *APOC3*, or in proximal genes [82]. *Sst*I polymorphism exists in a strong linkage disequilibrium with two functional polymorphisms located in the *APOC3* promoter region, c.-47-481T>C (rs2854117) and c.-47-454C>T (rs2854116) [6]. Both polymorphisms c.-47-481T>C and c.-47-454C>T are located in the insulin response element of *APOC3* promoter and have been shown to attenuate *APOC3* insulin responsiveness in vitro, and subsequently to increase production of apoC-III [82], which has been shown to cause a two-fold increase in fasting plasma TG concentration [83]. A study with heterozygous carriers for c.-47-481T>C and/or c.-47-454C>T reported an increase in fasting apoC-III plasma concentration and fasting TG plasma concentration and a reduction in the clearance of circulating TG [92]. In silico studies have predicted a post-transcriptional regulation mechanism of *APOC3* mRNA, where the single nucleotide substitution of *Sst*I polymorphism induces a potential loss in the binding of five microRNAs silencers endogenously expressed in the liver and intestine to its complementary *APOC3* 3′UTR sequence. However, this was not validated in vitro [93]. Another hypothesis was proposed, namely that an *Sst*I site located within 40 nucleotides from the 3′ polyA tail could inhibit the binding of specific proteins responsible for removing the poly-A tail, thus prolonging the half-life of *APOC3* mRNA [82].

Population studies reported that the promoter c.-47-639A>C (rs2542052) allele was associated with a decreased plasma concentration of apoC-III, suggesting a protective effect against CVD [94]. The Exome Sequencing Project [95] found an aggregate of rare LOF variants in the *APOC3* gene associated with lower plasma TG levels, namely missense variant c.127G>A (p.Ala43Thr) and three LOF variants: nonsense variant c.55C>T (p.Arg19*) and splice site variants c.55+1G>A (G to A transition in intron 2) and c.179+1G>T (G to T transversion in intron 3). Even though only a modest proportion of the population are carriers of these *APOC3* variants, TG plasma concentration in the identified heterozygous carriers were lower than levels in noncarriers, and CVD risk among carriers of any rare *APOC3* variant was 40% lower than the risk among noncarriers [95]. Similarly, another population-based study reported that LOF variants in *APOC3* were significantly associated with a reduction in postprandial TG concentration and in the risk of atherosclerotic diseases observed in heterozygous carriers [88].

## 7. Apolipoprotein A-V

Apolipoprotein A-V (apoA-V, (MIM*606368) plays both extracellular and intracellular roles in TG homeostasis, as it is an essential activator of LPL by stabilizing the lipoprotein-enzyme complex to enhance lipolysis. ApoA-V is predominantly expressed in the liver. Compared to other apoproteins, its plasma concentration is lower than plasma concentration of the most common apolipoproteins [96], with some estimations referring to one apoA-V molecule per 20–25 TRLs [97]. ApoA-V promotes TRL lipolysis by enhancing TRL adherence to the cell surface through HSPG and/or GPIHBP1. ApoA-V has also been shown to participate in the uptake of remnant particles and plays a role in hepatocyte VLDL secretion [96,97,98]. Based on these studies, an inverse relationship between apoA-V and plasma TG concentration is expected [99]. In fact, experimental models with mice overexpressing the gene via adenovirus vector transfection demonstrated a drastic reduction in TG concentration transported in the VLDL lipoprotein fraction compared to wild-type mice. Conversely, *apoa5* knockout mice demonstrated TG levels four times higher than wild-type mice [100]. ApoA-V is present in human serum at much lower concentrations than other apolipoproteins, and distribution was detectable in VLDLs, HDL and CMs [101].

ApoA-V is encoded by the *APOA5* gene, identified as an integral member of the *APOA1*-*APOC3*-*APOA4* genetic cluster located in the chromosomal region 11q23 [102]. The hepatic regulation mechanism of apoA-V and *APOA5* mRNA is not completely understood [103]. Several receptors are positively associated with the regulation of the *APOA5* gene, including PPARα, FXR, HNF4α and the orphan nuclear receptor Nur77. Conversely, the LXR factors, insulin and glucose, via the upstream stimulatory factor (USF), inhibit the expression of *APOA5* [99,104]. The *APOA5* gene comprises four exons and encodes for the 366 amino acid protein, of which 23 amino acids constitute the signal peptide. Cleavage of the signal peptide forms the mature apoA-V, a hydrophobic protein rich in α helices [96]. The N-terminal domain adopts an amphipathic helical conformation that binds to lipoproteins [99]. Next to the N-terminus is the central domain responsible for establishing ionic bonds with HSPG, cellular receptors R-LDL and LR11, and GPIHBP1 [99]. The cysteine at position 204 has the potential to form homodimers or heterodimers with plasma proteins through the formation of disulfide bonds [105]. Sun et al. further demonstrated that residues 192 to 238 are necessary for lipid binding and activation of LPL [106]. The C-terminal domain is composed of four consecutive proline amino acids important for lipid binding [98,103].

Table 4 describes the genetic variants in the *APOA5* gene associated with HTG. Five common polymorphisms are predominately inherited in three haplotypes: the APOA5*1 haplotype, the APOA5*2 haplotype and the APOA5*3 haplotype. The APOA5*1 haplotype is solely composed of wild-type alleles. The APOA5*2 haplotype is characterized by the presence of four polymorphisms: c.*158T>C (rs2266788, previously known as c.1891T>C, c.1259T>C, or SNP1), c.162-43A>G (rs2072560, previously known as IVS3+476G>A or SNP2), c.-72-571T>C (rs662799, previously known as g.-1131T>C or SNP3), and c.-3A>G (rs651821). These polymorphisms have been shown to coexist in 100% linkage disequilibrium, meaning they only occur in the combination described as the common allele c.-72-571T/-3A/*158T (TAT) or as the rare allele c.-72-571C/-3G/*158C (CGC) [104]. The APOA5*3 haplotype is characterized by the c.56C>G (p.Ser19Trp) polymorphism (rs3135506, also known as S19W).

Single nucleotide polymorphisms cause amino acid substitution and a subsequent morphological modification of the apoA-V molecule. Consequently, carriers express a functionally compromised or completely dysfunctional apoA-V [98]. The c.56C>G polymorphism replaces the serine amino acid at position 19 with a tryptophan (p.Ser19Trp) and affects signal peptide function, which compromises the efficiency of protein translocation across the ER, inducing 50% less secretion in vitro [107]. The c.−3A>G polymorphism located in the Kozak sequence, a highly conserved sequence in eukaryotes that precedes the AUG initiation codon, may reduce the expression by affecting translation initiation efficiency [108]. The c.553G>T polymorphism (rs2075291) causes the substitution of a glycine for a cysteine at position 185 (p.Gly185Cys) in exon 4, which increases the chances of intramolecular disulfide bond formation with cysteine 204. Given that this residue is located in a functional region responsible for establishing electrostatic interactions with HSPG and cellular receptors, the conformational change induced in this domain could compromise TRL lipolysis and/or clearance [108].

Several population studies have evaluated the relationship between *APOA5* polymorphisms, TG plasma concentration and atherosclerotic cardiovascular risk, with c.-72-571T>C and p.Ser19Trp being positively associated with HTG. Only the c.-72-571C allele was shown to directly affect apoA-V plasma concentration, leading to the enhanced synthesis of atherogenic LDL particles and arterial stiffness [109]. To explore the relationship with coronary artery disease (CAD) and variants c.-72-571T>C and p.Ser19Trp, a study examined participants with angiographically defined CAD (669 CAD and 244 CAD-free). Despite the observed association with an at-risk lipid profile, no significant difference was detected in the distribution of both *APOA5* gene polymorphisms between CAD and CAD-free defined subjects [110]. A different study that examined patients with or without angiographically defined CAD was also unsuccessful in providing a significant correlation between the p.Ser19Trp and p.Gly185Cys variants and cardiovascular risk [111].

*APOA5* variants may play a role in the individual sensitivity of circulating lipids from diet, as suggested by several works. One study examined a Puerto Rican population, aiming to determine the association of the genetic variants with plasma lipid concentrations, and detected significant interactions between total dietary fat intake as a percentage of total energy intake and *APOA5* polymorphisms associated with plasma TG and total cholesterol concentrations. This suggested that carriers of the variants may benefit from a low-fat diet to potentially result in a more atheroprotective lipid profile [112]. Another study examining the Mediterranean population found a significant genotype–dietary fat interaction for obesity traits and TRLs, replicating previous observations of gene–diet interactions between *APOA5* variants and fat intake [113]. Taken together, these population-based studies demonstrate how *APOA5* polymorphisms that alter apoA-V plasma levels or protein structure/function give rise to critical changes in lipid metabolism and ultimately potential susceptibility to cardiometabolic conditions. However, population-based results are conflicting and mostly reflect the difficulty in interpreting lipid metabolism studies, given the influence of other metabolic, dietary and genetic variables [97].

Since the discovery of the *APOA5* gene, over 400 genetic variants have been described (ClinVar: https://www.ncbi.nlm.nih.gov/clinvar, accessed on 20 December 2023). Priore Oliva et al. [114] reported the nonsense variant c.442C>T (p.Gln148* or p.Q148*), associated with chylomicronemia onset. The C → T transition in exon 4 introduces a premature termination codon, generating a truncated variant deficient in both the C-terminal lipid binding domain and the essential positively charged central domain necessary for binding to the GPIHBP1/HSPG/LDL receptor family. The proband presented severe HTG, eruptive xanthomatosis and decreased LPL activity. He was a homozygous carrier of the p.Gln148* (XX) variant, c.56C>G (p.Ser19Trp) (GG) and c.-72-571T>C (TT) polymorphisms in the *APOA5* gene, and c.-47-454C>T polymorphism (TT) in the *APOC3* gene [114]. Marcais et al. [115] demonstrated that individuals carrying the p.Gln139* variant exhibited severe chylomicronemia due to a significant LPL activity defect, and provided evidence supporting the functional interplay between apoA-V and LPL. p.Gln139* is predicted to determine a truncation at residue 116 of the mature protein. Priore Oliva et al. [116] described the c.161+3G>C variant, involving a G → C transversion at the donor splice site within intron 3. The *APOA5* variant led to the aberrant splicing of pre-mRNA, resulting in the exclusion of exon 3 and the introduction of a premature termination codon. Thus, variants described in the *APOA5* gene determine structural and functional changes in apoA-V, associated with HTG onset. However, the intervention of other genetic (polymorphisms) and non-genetic factors may be necessary for phenotypic expression [99]. GWAS findings indicate that genetic loci linked to lipid metabolism account for less than 10–12% of lipid variability [117]. As a result, it has been suggested that additional significant genetic contributions may be attributed to epigenetic mechanisms. Epigenome-wide association studies (EWAS) described the correlation between DNA methylation in some cytosines and the concentration of circulating lipids. A small number of cytosines (8–10 CG dinucleotides) that positively correlated with plasma TG levels were successfully identified. Cytosine cg12556569 has been identified as being significantly associated with TG metabolism in EWAS [118]. Cytosine cg12556569 is located in the promoter region of *APOA5* and has been found to significantly correlate with the variant rs964184 (724C>G), a polymorphism located in the *ZNF259*-*APOA5* intergenic region. This cytosine also exhibits correlations with other SNPs within the APOA5 gene [rs662799 (−72-571T>C) and rs3135506 (p.Ser19Trp)] [119]. Caussy et al. further proposed that the onset of HTG in carriers of the APOA5*2 haplotype may involve post-transcriptional regulation by silencing microRNAs. The microRNA miR-485-5p demonstrated complementarity with the rare c.*158C allele, located in the 3′UTR sequence of the *APOA5* gene. The binding of miR-485-5p to the c.*158C sequence significantly reduced *APOA5* promoter activity [120].

## 8. Lipase Maturation Factor 1

The *LMF1* gene, located on chromosome 16p13.3, is responsible for coding LMF1, a protein that participates in the maturation of LPL and hepatic lipase (HL) [121]. Functioning as an endoplasmic reticulum (ER) chaperone, this protein comprises five transmembrane domains along with a crucial conserved C-terminal domain that is vital for the activation of lipases. The precise molecular mechanism through which LMF1 operates is not entirely understood. It is proposed that LMF1 might enhance LPL stability by interacting with ER chaperones aiding in the formation of disulfide bonds in newly synthesized LPL [33].

Table 5 describes the genetic variants in the *LMF1* gene associated with HTG. Two nonsense variants in the *LMF1* gene were identified in homozygous carriers of c.1317C>G (p.Tyr439* or p.Y439*) [122], and c.1391G>A (p.Trp464* or p.W464*) [123], respectively, with severe HTG, recurrent episodes of acute pancreatitis, and combined lipase deficiency (MIM#246650). The individual homozygous for p.Tyr439* exhibited xanthomas, partial lipodystrophy, and a notable 93% decrease in LPL activity. In contrast, the homozygous carrier of p.Trp464* displayed a 76% reduction in LPL activity. The phenotypic differences observed between the two variants may be attributed to the preservation of a higher number of amino acid residues in the C-terminal domain of the p.Trp464* variant compared to the p.Tyr439* variant [121]. Another homozygous nonsense variant c.697C>T (p.Arg233*) was identified in the *LMF1* gene. The carrier reported childhood-onset severe HTG [124].

## 9. Glycosylphosphatidylinositol-Anchored High-Density Lipoprotein-Binding Protein 1

GPIHBP1 plays a vital role in translocating LPL to the luminal surface of capillary endothelial cells, ensuring its secure positioning and facilitating the margination of TRLs for lipolytic processing to take place. GPIHBP1 is a member of the “LU” protein family (LU: lymphocyte antigen 6–urokinase-type plasminogen activator receptor (uPAR)), characterized by an abundance of cysteines. Cysteine residues create disulfide bonds, playing a crucial role in shaping the distinctive three-dimensional three-finger fold conformation of Ly6 proteins [125]. The GPIHBP1 protein anchors the LPL enzyme in capillary endothelial cells in a stoichiometric ratio of 1:1 [126], performs translocation of LPL to the capillary lumen and marginalization of lipid substrate to the capillary endothelium [125], and prevents the spontaneous *unfolding* of LPL [40]. GPIHBPI is encoded by the *GPIHBP1* gene located in chromosome 8q24.3 and expressed by the capillary endothelium [38]. Exon 2 encodes an intrinsically disordered N-terminal domain, rich in acidic residues (Glu and Asp) important for stabilizing LPL’s catalytic domain [127]. Exons 3 and 4 encode for an Ly6/uPAR domain and a hydrophobic GPI signal peptide at the C-terminal domain responsible for the covalent binding of a GPI membrane anchor [33,38].

The GPIHBP1 protein is subjected to post-translational modifications, such as N-glycosylation, O-sulfation and disulfide bond formation [38]. N-glycosylation is essential for intracellular trafficking of the GPIHBP1 protein [33]. The five disulfide bonds play a crucial role in the three-finger fold conformation of the LU domain. The tertiary structure of LU is essential for the binding of GPIHBP1 to LPL, as well as for interaction with apolipoproteins C-II and A-V and lipoproteins marginalized throughout the endothelial surface [128]. The acidic region on the GPIHBP1 N-terminal domain establishes electrostatic interactions with LPL at the HSPG binding sites, while the LU domain interacts with the PLAT domain located at the C-terminal of LPL [38]. Once bound to LPL, GPIHBP1 translocates the enzyme across the endothelial cellular layer. The LPL-GPIHBP1 complex induces the marginalization of LRT along the capillary endothelium and initiates intravascular hydrolysis of TG [40].

Variants in the *GPIHBP1* gene have been described in patients with FCS [129]. Homozygous carriers of *GPIHBP1* variants exhibit persistent chylomicronemia with early onset during childhood, and severe HTG. A significantly reduced concentration of the LPL enzyme and GPIHBP1 in serum defines GPIHBP1 deficiency (MIM*612757). The phenotypic expression of GPIHBP1 deficiency only occurs in homozygous or compound heterozygous carriers of *GPIHBP1* gene variants, while heterozygous carriers are normolipidemic [130]. Most *GPIHBP1* missense variants (Table 6) affect the cysteine residues involved in the formation of disulfide bonds fundamental to the three-dimensional three-finger fold conformation of the LU domain, namely c.194G>C (p.Cys65Ser), c.194G>A (p.Cys65Tyr), c.202T>G (p.Cys68Gly), c.203G>A (p.Cys68Tyr), c.202T>C (p.Cys68Arg), c.247T>C (p.Cys83Arg), c.266G>T (p.Cys89Phe), and c.329G>A (p.Cys110Val) [21,125,126]. Amino acid substitutions within close proximity to disulfide bond-forming cysteines also compromise GPIHBP1’s ability to bind LPL, such as c.344A>C (p.Gln115Pro) and c.331A>C (p.Thr111Pro) [130]. The presence of unpaired cysteines can induce the multimerization of GPIHBP1 variant proteins, rendering GPIHBP1 unavailable for the formation of the LPL-GPIHBP1 complex [131]. The *GPIHBP1 missense* variants c.239C>A (p.Thr80Lys) and c.523G>C (p.Gly175Arg) interfere with N-glycosylation and intracellular trafficking of GPIHBP1 destined for the plasma membrane, respectively [132]. Although the acidic region corresponds to 30% of the primary sequence of mature GPIHBP1, there are no variants in this region associated with the HTG onset described [38].

## 10. Hypertriglyceridemia and Pancreatitis

Moderately severe and severe presentations of AP are linked to diverse local and systemic complications, potentially resulting in transient or persistent organ failure, impacting the kidneys, the lungs, and the cardiovascular system [133]. In addition, recurrent AP can lead to chronic pancreatitis, which increases the risk of pancreatic cancer [134]. AP is a sudden inflammation of the pancreas that lasts from days to several weeks. It is a potentially fatal condition that requires emergency hospitalization. There is no specific treatment and only supportive care with pain control, intravenous hydration and enteral nutrition are available [134].

The etiology of AP is mostly related to massive alcohol consumption, biliary disease and HTG. In fact, it is widely accepted that severe HTG increases the risk for AP. Nevertheless, the exact pathophysiology and the underlying mechanism remains unclear [135]. The most commonly accepted theory suggests the excess hydrolysis of TG, via pancreatic lipases, into fatty acids (FA), which in high concentrations are harmful, leading to injury of the pancreatic acinar cells and capillaries [136,137]. These FA induce sustained elevation of [Ca^2+^], inhibit mitochondrial complexes and ATP production in pancreatic acinar cells [138]. Other studies have also indicated that FA decrease the HCO_3_^−^ and fluid secretion of pancreatic ductal cells and reduce the function of pancreatic ducts [139,140]. Furthermore, high FA concentrations induce cytokine release and tissue injury and respiratory, kidney, and cardiovascular failure [134].

In support of the concept that HTG can initiate pancreatic injury, the perfusion of an ex vivo isolated pancreas with unsaturated TG caused a large increase in serum FA, with the organ becoming hemorrhagic [141]. In other experimental works, TG delivered directly into the pancreas also induced hemorrhage, prevented by lipolysis inhibition [137]. These studies strongly support the theory that TG lipolysis underlies the severe AP phenotype observed during HTG. Another less accepted theory focuses on plasma hyperviscosity. In HTG, the concentration of CMs is elevated. The high serum TG increases blood viscosity, which impairs blood flow and results in pancreatic ischemia and acidosis [137]. The acidosis may increase the potential for trypsinogen activation and initiate or aggravate inflammation [135].

In a meta-analysis study, the effects of various serum TG concentrations on the severity and mortality of AP were compared. It was reported that HTG significantly elevated the odds ratio for severe AP when compared to patients with normal serum TG levels. In addition, HTG was linked to higher occurrence of pancreatic necrosis, organ failure, and mortality [14]. Moreover, existing clinical data from HTG-AP patients, when systematically reviewed, suggest that the severity of HTG-AP may be greater than the severity of AP from other etiologies [142].

## 11. Hypertriglyceridemia and Atherosclerotic Cardiovascular Disease

Epidemiologic and genetic studies have established TRLs and their remnants as important contributors to atherosclerotic cardiovascular disease [143,144]. The atherosclerotic risk associated with TRLs is related to the concentration of the atherogenic apoB-containing particles and enhanced by their TG content [145,146]. In the general population, individuals with HTG have a significantly higher risk of coronary heart disease (CHD), ischemic stroke, and mortality [143,147,148]. Several studies have demonstrated a relationship between plasma levels of TRLs and the risk of CHD [149,150,151], and Mendelian randomization analysis provided causal evidence for the role of TG-mediated pathways in CHD incidence [152,153,154].

The relationship between LPL variants and CVD risk was highlighted by several studies and variants in some genes that modulate LPL activity, and have also been associated with CVD events. Nevertheless, this relationship may result from higher TG levels being atherogenic or because LPL modulates other processes, such as HDL levels and function. Further support for TG-lowering variants in LPL and CVD risk came from a study by Ference et al. with participants enrolled in cohort or case–control studies between 1948 and 2017 [155]. The data supported the clinical benefits of lowering TG, although it may require concomitant ApoB lowering. HTG’s relationship with atherosclerosis can be explained by several mechanisms. First, TRLs, like other apoB-containing lipoproteins, are atherogenic. Moreover, HTG induces alterations in lipoprotein profile as the activity of cholesteryl ester transfer protein (CETP), responsible for exchanging TG for cholesterol esters between TRLs and TG-poor lipoproteins, is stimulated. Thus, cholesterol depletion of LDL and HDL particles is increased, with a reduction in particle size and cholesterol content. The resulting small-dense LDL particles are more atherogenic [156].

Independent of the degree of elevation of serum TG, both genetic and lifestyle factors are key players in HTG pathophysiology. As previously stated, primary severe HTG has both monogenic and polygenic determinants, though most cases are polygenic and frequently coexist with nongenetic conditions. Cumulatively, multiple genetic variants can increase the risk of HTG, whereas environmental and lifestyle factors can induce phenotypic expression in a genetically susceptible individual.

**Figure 1:**
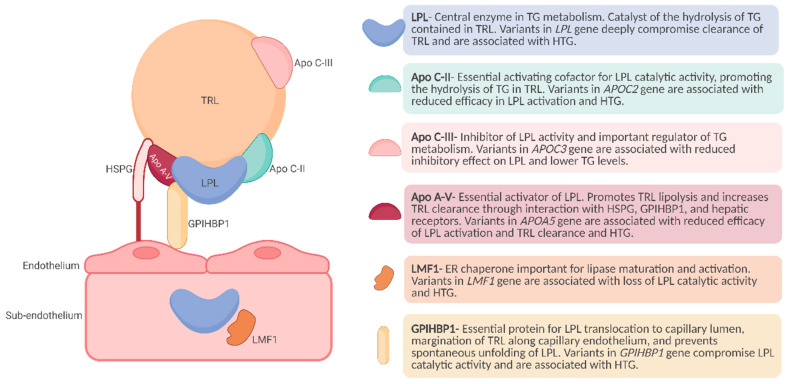
Lipoprotein lipase complex bound to endothelial cells. The lipolytic process of TRLs by LPL involves a complex interplay of multiple proteins. LPL is chaperoned by LMF1 during the biosynthesis pathway facilitating LPL maturation. After secretion, LPL binds to HSPG and is stabilized. LPL forms a complex with GPIHBP1, which is shuttled to the endothelial cell surface within the capillary lumen. The catalytic activity of LPL is promoted by ApoA-V and Apo C-II, while ApoC-III serves as an inhibitory factor. ApoC-II is crucial for the enzymatic activity and ApoA-V contributes to the stabilization of the LPL–TRL complex by interacting with HSPG and GPIHBP1. Genetic variants within the genomic locus responsible for regulating these key proteins in the lipolytic process can significantly compromise catalytic activity. LPL catalytic impairment impedes the efficient clearance of TRLs, ultimately leading to HTG. ApoA-V—apolipoprotein A-V; ApoC-II—apolipoprotein C-II; ApoC-III—apolipoprotein C-III; *APOA5*—Apo A-V gene; *APOC2*—ApoC-II gene; *APOC3*—ApoC-III gene; LPL—lipoprotein lipase; LMF1—Lipase maturation factor 1; GPIHBP1—glycosylphosphatidylinositol-anchored high-density lipoprotein-binding protein 1; ER—endoplasmic reticulum; HSPG—heparan sulfate proteoglycans; TG—triglycerides; TRLs—triglyceride-rich lipoproteins; HTG—hypertriglyceridemia. Figure icons were created with BioRender.com, accessed on 9 January 2024.

The major component of lipolytic removal of circulating TRLs is LPL. The enzyme, as already mentioned, is regulated by various apolipoproteins, namely APOC2, APOC3, APOA5, and angiopoietin-like proteins. Whereas APOC2 is an essential cofactor for LPL activity and APOA5 stabilizes the LPL–lipoprotein complex, APOC3 is believed to inhibit LPL activity. LMF1 promotes LPL maturation which is then transported transendothelially and bound to its anchoring protein GPIHBP1 on the luminal surface of the endothelium. Variants in these five canonical genes affect TG metabolism and may lead to HTG [157]. The intervention of these gene products in lipolysis is shown in Figure 1.

Variants in other genes may be involved in HTG mechanisms. Less common genes such as *LIPC* may also be associated with HTG. The *LIPC* gene is responsible for encoding HL, the enzyme involved in the hydrolysis of TG in remnants of TRLs and in the conversion of VLDLs to LDLs. It is a crucial enzyme in TG metabolism and a ligand/bridging factor for receptor-mediated lipoprotein clearance that is related to plasma TG and HDL-C levels [158]. In fact, HL is involved in the clearance of TG from VLDLs, and this function is dependent on the composition of HDL particles. Alterations in HDL-apolipoprotein composition can inhibit the release and the activation of the enzyme [159]. Alterations in HL activity have been associated with CAD. The effect on CAD risk is dependent on the underlying lipoprotein phenotype. Gene variants were associated with increased CAD risk in some population studies [160,161]. Increased HL is associated with smaller and denser LDL and HDL particles, while decreased HL is related to larger and more buoyant LDL and HDL particles. Central obesity with HTG is linked with high HL activity that leads to the formation of pro-atherogenic smaller and denser LDL [162].

## 12. Therapeutic Potential and Emerging Therapies

Individuals with severe HTG should receive TG-lowering pharmacotherapy, along with making lifestyle modifications. Treatment of these patients poses a huge challenge as traditional medicines have limited success in decreasing TG levels and/or in reducing the incidence of AP. Knowledge of physiopathological mechanisms may provide opportunities for targeted drug development. The genetic discoveries have facilitated the development of new pathway-specific therapeutics, and future research may reveal new candidate genes or targets. A current strategy is targeting LPL modulating proteins. Therapeutic options with novel mechanisms of action have been developed, such as antisense oligonucleotides (ASO) and small interfering RNA (siRNA) [163].

ApoC-III is an important target, as besides being a potent LPL inhibitor, it also presents LPL-independent mechanisms of increasing TG and TRLs. Therefore, apoC-III inhibitors have been the subject of several clinical trials. The apoC-III inhibitors include volanesorsen (an antisense oligonucleotide inhibitor of *APOC3* mRNA), olezarsen (an N-acetylgalactosamine-conjugated apolipoprotein C-III antisense oligonucleotide analogue), and ARO-APOC3 (siRNA).

Volanesorsen was the first ASO targeting *APOC3*. It blocks apo-CIII synthesis in hepatic cells by inhibiting *APOC3* mRNA, and has been approved by the European Medicines Agency (EMA) and the National Institute for Health and Care Excellence (NICE). Nevertheless, thrombocytopenia experienced by some patients remains the predominant concern necessitating close monitoring [164]. Olezarsen is an advanced form of volanesorsen since this ASO is conjugated with N-acetylgalactosamine, which presents binding affinity for the asialoglycoprotein type 1 receptor which enhances targeted delivery to hepatocytes. ARO-APOC3 is a GalNAc-conjugated siRNA that targets *APOC3* mRNA. Unlike ASO, which act in the nucleus of the hepatocyte, siRNA acts mainly in the cytoplasm. There are currently ongoing trials with ARO-APOC3 that will provide more information about this promising molecule [164].

Emerging TG-lowering therapies are also targeting ANGPTL3, as LOF variants are associated with decreased plasma TG and appear protective against CVD despite the lowering of HDL-C [165]. Several compounds have been developed, including a monoclonal antibody (mAb) against ANGPTL3 named evinacumab and an ASO named Vupanorsen. Nevertheless, these drugs were abandoned for HTG treatment [164]. A phase 2 trial with evinacumab was stopped in 2023 (sponsor’s decision, Regeneron) due to poor recruitment (NCT04863014) [166]. Vupanorsen, a GalNAc-conjugated ASO targeting *ANGPTL3* mRNA and thus inhibiting ANGPTL3 protein, causes hepatic fat and higher doses were associated with elevations in the liver enzymes ALT and AST. Therefore, drug development was discontinued in January 2022 [164]. ARO-ANG3, a siRNA targeting *ANGPTL3* is still under study, and early-phase data suggests that is generally well-tolerated [167].

Other studies related to targets directed towards the inhibition of ANGPTL4 are ongoing and may offer new avenues to reducing the risk of acute pancreatitis and CVD events [168].

## 13. Conclusions

Primary HTG has a complex genetic basis and presents an intricate genotype–phenotype correlation. Further studies are needed to investigate predictive and prognostic markers for pancreatic and cardiovascular complications, as the focus has been on reducing the risk of pancreatitis and the risk of CVD has been poorly investigated prospectively. There is an urgent need to address these issues. Genetic screening facilitates the identification of individuals who are at risk of CVD, directing them towards personalized pharmacological therapies and early lifestyle adjustments. Moreover, knowing the molecular diagnosis aids in the early identification of at-risk family members. Nevertheless, besides biallelic rare variants causing monogenic chylomicronemia, genetic determinants are not absolute indicators for HTG causality, as many of the variants only provoke the condition when other factors are present, such as diet, alcohol, medications, and diseases like diabetes and hypothyroidism.

Even though heterozygous rare variants and excess SNP accumulation are generally overrepresented in hypertriglyceridemic subjects, there is substantial overlap of risk allele scores between HTG and non-HTG affected individuals and high TG risk scores fail to provide conclusive evidence. Therefore, genomic tests are required to evaluate interactions between heterozygous variants and the SNP cumulative burden. Additionally, variants in non-canonical TG-regulating genes could also be explored.

Patients with FCS and MCM suffer from high morbidity and mortality with a significant loss of quality of life associated with severe HTG such as abdominal pain, episodes of potentially fatal AP, and psychosocial problems. Given the risks associated with HTG, a definitive identification and diagnosis is crucial in order to prevent the clinical complications associated with extremely high concentrations of plasma TG.

## Figures and Tables

**Table 1:** Genetic variants in the *LPL* gene associated with HTG.

Variant ID	Location	*LPL* Variant	Consequence	Type
rs1801177	Exon 2	c.106G>A	p.Asp36Asn ~85% of LPL secretion rate	Missense
rs118204057	Exon 5	c.644G>A	p.Gly215Glu Catalytically defective LPL	Missense
rs118204060	Exon 5	c.701C>T	p.Pro234Leu Catalytically defective LPL	Missense
rs118204068	Exon 6	c.829G>A	p.Asp277Asn Catalytically defective LPL	Missense
rs268	Exon 6	c.953A>G	p.Asn318Ser Partial LPL catalytic activity Low secretion rate of LPL	Missense
rs1800590	Promoter	c.-188-93T>G	Increased *LPL* promoter activity 18–24% (LD with p.Asp36Asn)	Regulatory
rs328	Exon 9	c.1421C>A	p.Ser474* Potential loss of miRNA binding site	Nonsense
rs1800011	Exon 6	c.862G>A	p.Ala288Thr ~80% of LPL secretion rate	Missense
______	Intron 8	c.1322+1G>A	Aberrant splicing and alternative transcripts	Splicing
CR951556 (HGMD)	Promoter	c.-188-39 T>C	Loss of transcription factor Oct-1 binding site Inhibits *LPL* promoter activity by 85%	Regulatory
CM941049 (HGMD)	Exon 2	c.209A>G	p.Asn70Ser N-glycosylation	Missense
______	Exon 5	c.615T>A	p.Cys243Ser Disulfide bond	Missense
rs118204082	Exon 6	c.798C>G	p.Cys266Trp Disulfide bond	Missense
rs781614031	Exon 5	c.547G>A	p.Asp183Asn Catalytic triad	Missense
rs118204064	Exon 5	c.548A>G	p.Asp183Gly Catalytic triad	Missense
rs781614031	Exon 5	c.547G>C	p.Asp183His Catalytic triad	Missense
rs191402029	Exon 5	c.542G>A	p.Gly181Ser Close to catalytic triad	Missense
rs118204062	Exon 6	c.809G>A	p.Arg270His Close to catalytic triad	Missense
CM962613 (HGMD)	Exon 6	c.809G>T	p.Arg270Leu Close to catalytic triad	Missense
rs587777909	Exon 8	c.1306G>A	p.Gly436Arg Furin cleavage	Missense
CM941058 (HGMD)	Exon 8	c.1310A>T	p.Glu437Val Furin cleavage	Missense
rs1296226558	Exon 8	c.1211T>G	p.Met404Arg Abolishes LPL-GPIHBP1 bond	Missense
rs118204079	Exon 9	c.1334G>A	p.Cys445Tyr Abolishes LPL-GPIHBP1 bond	Missense
CM040449 (HGMD)	Exon 5	c.602A>T	p.Asp201Val Abolishes LPL-GPIHBP1 bond	Missense

Variants from dbSNP are identified by using a reference SNP (rs) number, a unique identifier assigned to a specific SNP (single nucleotide polymorphism). Variants from HGMD are identified by using accession numbers that are specific to the HGMD database. LPL—lipoprotein lipase; GPIHBP1—glycosylphosphatidylinositol-anchored high-density lipoprotein-binding protein 1; LD—linkage disequilibrium.

**Table 2:** Genetic variants in the *APOC2* gene associated with HTG.

Variant ID	Location	*APOC2* Variant	Consequence	Type
rs120074116	Exon 4	c.255C>A (apoC-II_Auckland_)	p.Tyr85*	Nonsense
rs120074111	Exon 3	c.177C>G (apoC-II_Bari_)	p.Tyr59*	Nonsense
rs1430203751	Exon 3	c.133_134del (apoC-II_Colombia_)	p.Ser45Glnfs*24	Frameshift
rs368487465	Exon 3	c.118del (apoC-II_Nijmegen_)	p.Val40*	Frameshift
rs120074111	Exon 3	c.177C>A (apoC-II_Padova_)	p.Tyr59*	Nonsense
CX160305 (HGMD)	Exon 3	c.86delinsCC (apoC-II_Shangai_)	p.Asn29Alafs*2	Frameshift
CD880084 (HGMD)	Exon 4	c.270del (apoC-II_Toronto_)	p.Asp69Thrfs*7	Frameshift
rs202190413	Exon 2	c.10C>T (apoC-II_Paris2_)	p.Arg4*	Nonsense
rs120074112	Exon 2	c.1A>G (apoC-II_Paris1_)	p.?	Missense
rs111628497	Intron 2	c.55+1G>C (apoC-II_Hamburg/Tokyo_)	Aberrant splicing and alternative transcripts	Splicing
______	Intron 1	g.17,719,326_17,722,303del (apoC-II_Tuzla_)	Deletion of exons 2, 3 and 4	Deletion
______	Promoter	c.-25-90A>G	Loss of transcription factor binding site Decreased *APOC2* promoter activity	Regulatory
______	Promoter	c.-25-190T>A	Loss of transcription factor binding site Decreased *APOC2* transcriptional activity	Regulatory

Variants from dbSNP are identified by using a reference SNP (rs) number, a unique identifier assigned to a specific SNP (single nucleotide polymorphism). Variants from HGMD are identified by using accession numbers that are specific to the HGMD database. apoC-II—apolipoprotein C-II; *APOC2*—apoC-II gene.

**Table 3:** Genetic variants in the *APOC3* gene associated with HTG.

Variant ID	Location	*APOC3* Variant	Consequence	Type
rs5128	3’UTR	c.*40G>C (Sst l)	Potential loss of miRNA binding site Increased *APOC3* promoter activity	Regulatory
rs2854117	Promoter	c.-47-481T>C	Loss of transcription factor insulin binding site Increased *APOC3* promoter activity (LD with c.*40G>C)	Regulatory
rs2854116	Promoter	c.-47-454C>T	Loss of transcription factor insulin binding site Increased *APOC3* promoter activity (LD with c.*40G>C)	Regulatory
rs2542052	Promoter	c.-47-639A>C	Decreased *APOC3* promoter activity	Regulatory
rs147210663	Exon 2	c.127G>A	p.Ala43Thr	Missense
rs76353203	Exon 1	c.55C> T	p.Arg19*	Nonsense
rs138326449	Intron 2	c.55+1G>A	Aberrant splicing	Splicing
rs140621530	Intron 3	c.179+1G>T	Aberrant splicing and alternative transcripts	Splicing

Variants from dbSNP are identified by using a reference SNP (rs) number, a unique identifier assigned to a specific SNP (single nucleotide polymorphism). Variants from HGMD are identified by using accession numbers that are specific to the HGMD database. *APOC3*—apo C-III gene; LD—linkage disequilibrium.

**Table 4:** Genetic variants in the *APOA5* gene associated with HTG.

Variant ID	Location	*APOA5* Variant	Consequence	Type
rs2266788	3′UTR	c.*158T>C (SNP1)	APOA5*2 haplotype	Regulatory
rs2072560	Intron 3	c.162-43A>G (SNP2)	APOA5*2 haplotype	Regulatory
rs662799	Intergenic region	c.-72-571T>C (SNP3)	APOA5*2 haplotype	Regulatory
rs651821	Intron 1	c.-3A>G	APOA5*2 haplotype Kozak sequence Reduced translation initiation efficiency	Regulatory
rs3135506	Exon 3	c.56C>G	p.Ser19Trp 50% secretion rate	Missense
rs2075291	Exon 4	c.553G>T	p.Gly185Cys Intramolecular disulfide bond	Missense
CM050179 (HGMD)	Exon 4	c.442C>T	p.Gln148*	Nonsense
rs121917821	Exon 4	c.415C>T	p.Gln139*	Nonsense
rs372791079	intron 3	c.161+3G>C	Aberrant splicing and alternative transcripts	Splicing

Variants from dbSNP are identified by using a reference SNP (rs) number, a unique identifier assigned to a specific SNP (single nucleotide polymorphism). Variants from HGMD are identified by using accession numbers that are specific to the HGMD database. *APOA5*—apolipoprotein A-V gene.

**Table 5:** Genetic variants in the *LMF1* gene associated with HTG.

Variant ID	Location	*LMF1* Variant	Consequence	Type
rs121909397	Exon 9	c.1317C>G	p.Tyr439* 93% loss of LPL catalytic activity	Nonsense
rs587777626	Exon 9	c.1391G>A	p.Trp464* 76% loss of LPL catalytic activity	Nonsense
rs199953320	Exon 5	c.697C>T	p.Arg233*	Nonsense

Variants from dbSNP are identified by using a reference SNP (rs) number, a unique identifier assigned to a specific SNP (single nucleotide polymorphism). Variants from HGMD are identified by using accession numbers that are specific to the HGMD database. LPL—lipoprotein lipase; LMF1—lipase maturation factor 1.

**Table 6:** Genetic variants in the *GPIHBP1* gene associated with HTG.

Variant ID	Location	*GPIHBP1* Variant	Consequence	Type
rs587777638	Exon 3	c.194G>C	p.Cys65Ser Disulfide bond of LU domain	Missense
CM102481 (HGMD)	Exon 3	c.194G>A	p.Cys65Tyr Disulfide bond of LU domain	Missense
rs587777639	Exon 3	c.202T>G	p.Cys68Gly Disulfide bond of LU domain	Missense
CM102970 (HGMD)	Exon 3	c.202T>C	p.Cys68Tyr Disulfide bond of LU domain	Missense
______	Exon 3	c.203G>A	p.Cys68Arg Disulfide bond of LU domain	Missense
CM1610274 (HGMD)	Exon 3	c.247T>C	p.Cys83Arg Disulfide bond of LU domain	Missense
rs587777640	Exon 3	c.266G>T	p.Cys89Phe Disulfide bond of LU domain	Missense
rs1328400518	Exon 4	c.329G>A	p.Cys110Val Disulfide bond of LU domain	Missense
rs587777637	Exon 4	c.344A>C	p.Gln115Pro Close to Cys from disulfide bond of LU domain	Missense
rs587777641	Exon 4	c.331A>C	p.Thr111Pro Close to Cys from disulfide bond of LU domain	Missense
rs749374488	Exon 3	c.239C>A	p.Thr80Lys N-glycosylation	Missense
rs145844329	Exon 4	c.523G>C	p.Gly175Arg Trafficking	Missense

Variants from dbSNP are identified by using a reference SNP (rs) number, a unique identifier assigned to a specific SNP (single nucleotide polymorphism). Variants from HGMD are identified by using accession numbers that are specific to the HGMD database. GPIHBP1—glycosylphosphatidylinositol-anchored high-density lipoprotein–binding protein 1; LU domain—Ly6/uPAR domain.
